# Mortality predictors and survival nomogram for hospitalized diabetic foot patients: a decade-long cohort

**DOI:** 10.3389/fendo.2026.1688571

**Published:** 2026-02-25

**Authors:** Jiacheng Li, Jianyuan Shi, Junyi Gu, Huili Cai, Xueming Gu, Jie Yang, Zhengyi Tang, Weiqing Wang

**Affiliations:** 1 Department of Endocrine and Metabolic Diseases, Shanghai Institute of Endocrine and Metabolic Diseases, Ruijin Hospital, Shanghai Jiao Tong University School of Medicine, Shanghai, China; 2 Shanghai National Clinical Research Center for Metabolic Diseases, Key Laboratory for Endocrine and Metabolic Diseases of the National Health Commission of the PR China, Shanghai Key Laboratory for Endocrine Tumor, Ruijin Hospital, Shanghai Jiao Tong University School of Medicine, Shanghai, China; 3 Department of Internal Medicine, Shanghai Yuanyang Hospital, Shanghai, China

**Keywords:** diabetic foot ulcer, mortality rate, Kaplan-Meier analysis, prediction model, risk factor

## Abstract

**Objectives:**

To evaluate mortality and risk factors in moderate-to-severe diabetic foot ulcer (DFU) hospitalized patients and develop a prognostic tool.

**Methods:**

This cohort study enrolled 485 eligible DFU patients (2009-2014), followed through 2024. Mortality was analyzed using Cox regression and Kaplan-Meier methods, focusing on peripheral artery disease (PAD) and other risk factors (p<0.05). A nomogram predicting 3-year mortality was developed based on multivariate Cox analysis.

**Results:**

The 3-year all-cause mortality rate in this cohort of 485 diabetic foot ulcer patients was 49.3%. The two most salient predictors of mortality were renal impairment and PAD severity. Patients requiring dialysis had a 3.05-fold increased risk of death, while the risk escalated sharply with PAD severity, reaching a 3.57-fold increase for severe PAD. The prognostic nomogram, which integrated these key factors, demonstrated strong predictive accuracy for 3-year survival, with a C-index of 0.79 and a 3-year AUC of 0.87.

**Conclusions:**

Hospitalized patients with moderate-to-severe diabetic foot show high mortality, predominantly associated with dialysis, CKD, and PAD. The developed nomogram effectively predicts 3-year mortality risk.

## 1 Introduction

Diabetic foot ulcer (DFU) is a prevalent and severe complication of diabetes, representing the leading cause of non-traumatic, hard-to-heal amputations. The 5-year mortality rate for patients with DFU is 49.1%, with a mortality risk 2.5 times higher than that of diabetic patients without foot ulcers (1, 2). Globally, approximately 18.6 million people are affected by DFU annually (3). China has an estimated diabetes mellitus (DM) prevalence of 11.6% among the adult population (4, 5). Consequently, a significant proportion of patients in China are affected by diabetic foot.

Although existing studies have identified multiple risk factors—such as chronic kidney disease (CKD), peripheral artery disease (PAD), and infection—as being associated with mortality in diabetic foot patients, the combined impact of these factors and their long-term predictive ability in the Chinese population remain underexplored and warrant further investigation (6–10). Patients with DFU frequently present with concurrent conditions such as neuropathy, vascular disease, and other systemic complications. Additionally, the structural abnormalities in their feet are often irreversible, and the overall condition tends to persist, underscoring the critical need for long-term follow-up. Currently, only a limited number of studies have followed cohorts of diabetic foot patients for 5 years or longer to investigate risk factors for mortality, and there is a lack of predictive models for these risk factors (6, 7, 10–12). Research has demonstrated that the nomogram model excels in visually predicting adverse events and supporting clinical decision-making (13). Graphically representing the numerical relationship between specific diseases and risk factors through a scoring system—without the need for complex calculations—facilitates effective screening of high-risk patients and timely interventions (14).

We conducted a long-term cohort study to assess mortality and identify risk factors among hospitalized patients with moderate-to-severe DFUs, with the objective of developing an accurate prediction model. To our knowledge, this represents the first extended follow-up study of this duration specifically targeting the diabetic foot population in China, addressing a critical knowledge gap as previous research has predominantly examined ulcer progression and amputation risks while largely overlooking mortality - the most clinically significant endpoint for patients with advanced DFUs.

## 2 Methods

### 2.1 Subjects

From January 2009 to December 2014, a total of 981 patients hospitalized in our department for DFU were consecutively enrolled. Specifically, the inclusion criteria were as follows: (1) age ≥18 years at the time of admission; (2) confirmed diagnosis of diabetes mellitus (including both type 1 and type 2) or ongoing antidiabetic medication or insulin therapy; (3) diagnosis of diabetic foot (patients with high-risk factors for Wagner grade 0 diabetic foot were included as controls); (4) met the hospitalization conditions for diabetic foot (15). The exclusion criteria were: (1) presence of severe organ failure, Cushing’s syndrome, cancerous ulcers, or other life-threatening conditions; (2) history of DFU; (3) history of major amputation; (4) inability to complete follow-up or incomplete data. Following the selection process, 485 DFU patients were included in our cohort. The study protocol received approval from the Institutional Review Board, with a granted waiver of informed consent.

### 2.2 Assessment for mortality

The individuals in the cohort were followed up, and the event time (mortality rate) was calculated from the date of study entry to the date of death or the last follow-up until December 2024 (whichever occurred earlier). Participants or their families were contacted via telephone. If follow-up was missed on three consecutive occasions, the case was recorded as lost to follow-up. In cases where the lack of follow-up was due to the patient’s death, the date and cause of death were obtained and entered into the dataset. The primary focus of the follow-up included wound healing and recurrence after discharge, as well as whether the patient had undergone amputation recently.

### 2.3 Measurements and definitions

All enrolled patients underwent routine interviews and examinations within 24 hours of admission to collect baseline information, including: the type of diabetes, disease duration, and previous and current treatment status; the occurrence of foot ulcers; and the presence of a history of hypertension, coronary heart disease, heart failure, chronic kidney disease, dialysis, stroke, or smoking. All necessary auxiliary examinations must be completed within 48 hours of admission, including but not limited to: fasting blood glucose (FBG), albumin (ALB), triglycerides (TG), total cholesterol (TC), high-density lipoprotein cholesterol (HDL-C), low-density lipoprotein cholesterol (LDL-C), white blood cells (WBC), and C-reactive protein (CRP). The ulcer conditions were diagnosed by the multidisciplinary expert team at the research center, and their Wagner grade, infection grade, ischemia grade, and PAD grade were recorded.

All laboratory parameters were measured from fasting venous blood samples collected at admission. FBG was determined by the glucose oxidase method, while glycated hemoglobin (HbA1c) was quantified using high-performance liquid chromatography (HPLC) standardized according to the National Glycohemoglobin Standardization Program (NGSP) (reference range <6.5%). Serum ALB was measured via the bromocresol green method. The lipid profile, including TG, TC, HDL-C, and LDL-C, was analyzed using enzymatic colorimetric assays. CRP levels were determined by immunoturbidimetry to assess inflammatory status. Complete blood count parameters—hemoglobin (Hb), WBC, and platelet count (PLT)—were analyzed using an automated hematology analyzer, with anemia defined per World Health Organization criteria as Hb <130 g/L for men and <120 g/L for women.

CKD was defined as an estimated glomerular filtration rate (eGFR) of <60 mL/min/1.73 m², corresponding to CKD stages G3-G5 according to the KDIGO classification. This threshold was selected to specifically evaluate the impact of moderate-to-severe renal impairment on clinical outcomes in diabetic foot patients. Dialysis was defined as patients who had undergone dialysis therapy, with their eGFR typically below 15 mL/min/1.73 m², primarily representing end-stage renal disease (ESRD).

The ulcer depth of the patients was classified according to the Wagner classification: Grade 0 (pre-ulcer or post-ulcer lesion), Grade 1 (partial or full-thickness ulcer), Grade 2 (probe to tendon or capsule), Grade 3 (osteomyelitis), Grade 4 (partial foot gangrene), and Grade 5 (full-foot gangrene). During the subsequent analysis, patients with Wagner grades 0–3 were classified into the low Wagner group, while those with grades 4–5 were classified into the high Wagner group. For clinical relevance, Wagner grades 4-5 (characterized by gangrene) were considered representative of moderate-to-severe DFU in this study, consistent with their pathophysiological severity.

The infection status of patients was classified according to the Infectious Diseases Society of America (IDSA) classification system (16): Grade 1 (no clinical manifestations of infection), Grade 2 (infection limited to the skin and subcutaneous tissue), Grade 3 (infection extending >2 cm or involving deep tissues), and Grade 4 (infection accompanied by systemic toxic symptoms). In the subsequent analysis, patients with infection grades 1-2 were classified into the low infection group, while those with grades 3-4 were classified into the high infection group.

The ischemia status of the patients was categorized based on the ankle-brachial index(ABI) (17, 18), with supplementary assessments performed using bifunctional ultrasound or angiography. The severity of PAD was further classified according to the ABI values as follows: none (ABI >0.9), mild (ABI 0.7–0.9), moderate (ABI 0.41–0.69), or severe (ABI ≤0.4) (19, 20).

In this study, multiple amputations were defined as patients undergoing two or more lower extremity amputation surgeries, either at different time points or simultaneously, due to infection, ischemic necrosis, or other pathological reasons. The amputation sites were limited to the lower limbs, including but not limited to the toes, soles of the feet, ankles, calves, and thighs.

### 2.4 Statistical analysis

Baseline variables were described according to their distribution using mean, standard deviation (SD), range, or frequency tables. The Cox proportional hazards regression model was employed to analyze potential risk factors or confounding factors associated with mortality outcomes. Missing events were defined as patients who remained alive or were lost to follow-up by the end of the study in December 2024. The most significant risk factors were graphically evaluated using Kaplan-Meier curves. The following factors were included in the analysis as potential predictors or confounders: age, gender, presence or absence of PAD, multiple amputations, history of dialysis, chronic kidney disease, history of coronary heart disease, history of heart failure, history of stroke, hypertension, history of smoking, Wagner group, infection group, type of diabetes, duration of diabetes, FBG, ALB, TG, TC, HDL-C, LDL-C, WBC, and CRP. Candidate variables with a univariate association of p < 0.10 were considered for the multivariable Cox model. The final predictors were determined through a consensus approach combining bidirectional stepwise selection (with a significance level of p < 0.05 for entry and retention) and Least Absolute Shrinkage and Selection Operator (LASSO) regression. Variables consistently selected by both methods were retained in the final model to ensure robustness and mitigate overfitting. No significant violation of the Proportional Hazards assumption was detected (based on Schoenfeld residual tests) for the variables in the final model. In the final regression model, two factors with a large sample size and high risk were selected for stratified analysis, and the corresponding Kaplan-Meier curves were plotted.

Based on significant risk factors identified through Cox multiple regression analysis, we developed a nomogram prediction model to assess 3-year mortality risk in hospitalized diabetic foot patients. The model assigns weighted scores to each variable based on standardized regression coefficients. During validation, we evaluated model discrimination using Harrell’s C-index (95% CI) and constructed time-dependent receiver operating characteristic (ROC) curves to determine the 3-year AUC value. Internal validation was performed using bootstrap resampling (500 repetitions) to calculate the calibration slope and shrinkage factor after optimism correction, with calibration curves comparing predicted versus observed 3-year survival probabilities. Additionally, we assessed the proportion of outcome variation explained by the model using corrected R² and evaluated model generalizability by comparing C-index differences (ΔC-index) between training and validation sets. All validations employed a closed-loop cross-validation strategy, with continuous variables incorporated in their original scale to preserve information. Model performance thresholds were established as follows: C-index >0.7 indicates clinical utility, and calibration slopes between 0.9-1.1 were considered acceptable.

All statistical analyses were performed using R software (version 4.4.1; R Foundation for Statistical Computing, Vienna, Austria), utilizing packages including survival, rms, glmnet, timeROC, boot, survivalROC, and pROC. Unless otherwise specified, all statistical tests were two-sided and conducted at a significance level of 5%.

## 3 Results

The median follow-up duration was 3.1 years (IQR: 1.3-10.8 years), encompassing both survivors and deceased patients. The resulting cumulative observation period was 3,193 person-years. During the ten-year follow-up period, a total of 91 cases were lost to follow-up.

### 3.1 Baseline characteristics

**Table 1** summarizes the baseline characteristics of the study participants included in the univariate analysis and Cox multiple regression model, as well as the association between each variable and mortality. Among the study participants, 12 patients (2.5%) were diagnosed with type 1 diabetes mellitus, while the remaining 473 cases (97.5%) were classified as type 2 diabetes mellitus.

**Table 1:** The baseline characteristics of the participants and the relationship between variables and death in univariate analysis and Cox multiple regression models.

Variable	All (n = 485)	Univariate analysis and Cox multiple regression models					
		Univariate analysis	P-value	Model 1	P-value	Model 2	P-value
Age (years) [mean (SD)]	64,4(12.3)	1.07(1.06-1.08)	<0.001^*^	1.07(1.06-1.09)	<0.001^*^	1.07(1.06-1.08)	<0.001^*^
Male gender [n (%)]	290(59.7)	1.31(1.05-1.63)	0.016^*^	1.34(1.07-1.68)	0.012^*^	1.33(1.06-1.67)	0.014^*^
Type 2 diabetes [n (%)]	473(97.5)	2.37(0.98-5.73)	0.056	——		——	
Multiple amputations [n (%)]	118(24.3)	2.17(1.71-2.75)	<0.001^*^	1.46(1.12-1.90)	0.005^*^	1.27(0.96-1.65)	**0.097**
Dialysis [n (%)]	29(5.9)	4.11(2.77-6.08)	<0.001^*^	2.52(1.57-4.05)	<0.001^*^	3.05(1.88-4.95)	<0.001^*^
CKD [n (%)]	88(18.1)	2.24(1.74-2.90)	<0.001^*^	2.63(1.91-3.61)	<0.001^*^	2.55(1.85-3.52)	<0.001^*^
History of CHD [n (%)]	69(14.2)	2.07(1.57-2.73)	<0.001^*^	1.67(1.26-2.23)	<0.001^*^	1.68(1.26-2.23)	<0.001^*^
History of stroke [n (%)]	63(12.9)	1.62(1.21-2.18)	<0.001^*^	1.77(1.30-2.40)	<0.001^*^	1.50(1.10-2.05)	0.010^*^
Heart failure [n (%)]	106(21.8)	2.01(1.58-2.56)	<0.001^*^	——		——	
Smoking [n (%)]	252(51.9)	1.22(0.99-1.51)	0.063	——		——	
PAD [n (%)]	391(80.6)	1.74(1.30-2.32)	<0.001^*^	1.73(1.28-2.33)	<0.001^*^	——	
PAD mild [n (%)]	97(20.0)	——		——		1.18(0.82-1.70)	**0.376**
PAD moderate [n (%)]	224(46.1)	——		——		1.82(1.32-2.50)	<0.001^*^
PAD severe [n (%)]	70(14.4)	——		——		3.57(2.43-5.27)	<0.001^*^
Diabetes duration (years) [mean (SD)]	12.5(8.7)	1.02(1.01-1.03)	<0.001^*^	——		——	
Hypertension [n (%)]	329(67.8)	1.39(1.10-1.76)	0.006^*^	——		——	
High Wagner group [n (%)]	218(44.9)	1.70(1.37-2.10)	<0.001^*^	——		——	
High Infection group [n (%)]	240(49.4)	1.56(1.26-1.94)	<0.001^*^	1.49(1.18-1.88)	<0.001^*^	1.40(1.11-1.76)	0.005^*^
FBG (mmol/L) [mean (SD)]	8.8(2.6)	1.005(0.965-1.046)	0.812	——		——	
HbA1c (%) [median (IQR)]	9.0(7.6-10.5)	0.989(0.942-1.039)	0.667	——		——	
ALB (g/L) [mean (SD)]	32.6(5.5)	0.983(0.964-1.002)	0.088	——		——	
TG (mmol/L) [mean (SD)]	1.3(0.5)	1.02(0.84-1.25)	0.834	——		——	
TC (mmol/L) [mean (SD)]	4.0(1.0)	0.995(0.895-1.107)	0.932	——		——	
HDL-C (mmol/L) [mean (SD)]	1.0(0.5)	0.70(0.55-0.89)	0.003^*^	——		——	
LDL-C (mmol/L) [mean (SD)]	2.7(0.9)	0.94(0.83-1.06)	0.297	——		——	
WBC (10^9^/L) [mean (SD)]	9.1(3.9)	1.018(0.992-1.045)	0.176	——		——	
Hemoglobin (g/L) [median (IQR)]	108.6 (95.9-121.9)	0.993(0.989-0.998)	0.004^*^	——		——	
Platelet (10^9^/L) [mean (SD)]	234.2(97.1)	1.000(0.999-1.001)	0.631	——		——	
CRP (mg/L) [mean (SD)]	46.6(52.0)	1.003(1.001-1.005)	0.002^*^	——		——	

CKD, chronic kidney disease; CHD, Coronary Heart Disease; PAD, Peripheral Arterial Disease; FBG, Fasting blood glucose; ALB, albumin; TG, triglyceride; TC, total cholesterol; HDL-C, high density lipoprotein cholesterol; LDL‐C, low density lipoprotein cholesterol; WBC, white blood cells; CRP, C-reaction protein.

HRs reported to 2 decimal places, except for values near null (0.990-1.010) where 3 decimal places are shown to preserve effect size accuracy.

Data are HRs (95% CIs).

Values in bold are P-values for risk factors from the two multivariable regression models.

∗P<0.05 (statistically significant for the risk factor in the model).

Univariate analysis revealed that dialysis and CKD were the most significant prognostic predictors in DFU patients (both p<0.001). While diabetes classification and smoking history demonstrated an elevated risk trend (HR>1), these associations were not statistically significant (p>0.05). The Cox multiple regression model (Model 1), constructed following variable screening, further confirmed dialysis and CKD as core predictive indicators. However, after performing stratified analysis for PAD (Model 2), the results demonstrate that dialysis and severe PAD constitute the most significant risk factors.

### 3.2 Mortality rate

As of December 31, 2024, among the 485 patients included in the cohort study, a total of 342 deaths were recorded. Among these, 172 cases (50.3%) died from heart disease, 50 cases (14.6%) from renal failure, 34 cases (9.9%) from stroke, 26 cases (7.6%) from sepsis, and 19 cases (5.6%) from tumors. Thirteen cases (3.8%) died from COVID-19, and the cause of death remained unknown for 28 cases (8.2%).

To further evaluate the long-term impact of PAD on patient mortality, we analyzed the 1-, 3-, 5-, and 10-year mortality rates of all patients, as well as those with and without PAD (**Table 2**). The results demonstrated that the mortality rate of patients with PAD was significantly higher than that of patients without PAD, with this difference exhibiting a distinct temporal trend during the follow-up period. Stratified analysis revealed that the mortality rate of patients with PAD was significantly higher than that of patients without PAD at 1, 3, 5, and 10 years. Notably, the difference in mortality rates between patients with and without PAD peaked at 3 years, followed by 5 years, 1 year, and 10 years.

**Table 2:** Cumulative probabilities (in percentages) of death.

Patient classification	Year 1		Year 3		Year 5		Year 10	
	Value	95% CI	Value	95% CI	Value	95% CI	Value	95% CI
All patients	20.2	16.6-23.8	49.3	44.8-53.7	61.9	57.5-66.2	70.5	66.5-74.6
Patients without PAD	7.4	5.1-9.8	30.9	26.7-35.0	46.8	42.4-51.2	58.5	54.1-62.9
Patients with PAD	23.2	19.5-27.0	53.7	49.2-58.1	65.5	61.2-69.7	73.4	69.5-77.3
Patients with moderate PAD and no CKD	14.0	9.6-19.9	47.8	40.6-55.2	58.6	51.2-65.7	66.7	59.4-73.4
Patients with moderate PAD and CKD	44.7	29.1-61.3	76.3	60.0-87.5	86.8	71.7-94.9	92.1	78.1-97.8
Patients with severe PAD and no CKD	46.4	33.7-59.6	82.1	69.6-90.5	89.3	77.8-95.3	92.9	82.3-97.4
Patients with severe PAD and CKD	78.6	49.2- 95.3	85.7	57.2- 98.2	100.0	76.8-100.0	100.0	76.8-100.0

### 3.3 Association analysis

In the multivariate regression analysis, two models were constructed to evaluate independent risk factors for mortality. In the first model, all selected factors demonstrated statistical significance (p < 0.05). Among these, dialysis (HR 2.52 [95% CI, 1.57–4.05]) and CKD (HR 2.63 [1.91–3.61]) exhibited the highest HR, indicating that these factors contributed particularly significantly to patient mortality risk. To further assess the impact of PAD severity on mortality risk, a stratified analysis of PAD was conducted in the second model, categorizing PAD into mild, moderate, and severe. The results revealed that multiple amputations, history of stroke, and mild PAD were no longer statistically significant compared to the first model (p > 0.05). Furthermore, in the second model, moderate PAD (HR 1.82 [1.32–2.50]) and severe PAD (HR 3.57 [2.43–5.27]) remained significant (p < 0.05).

### 3.4 Kaplan-Meier survival curves

**Figure 1:**
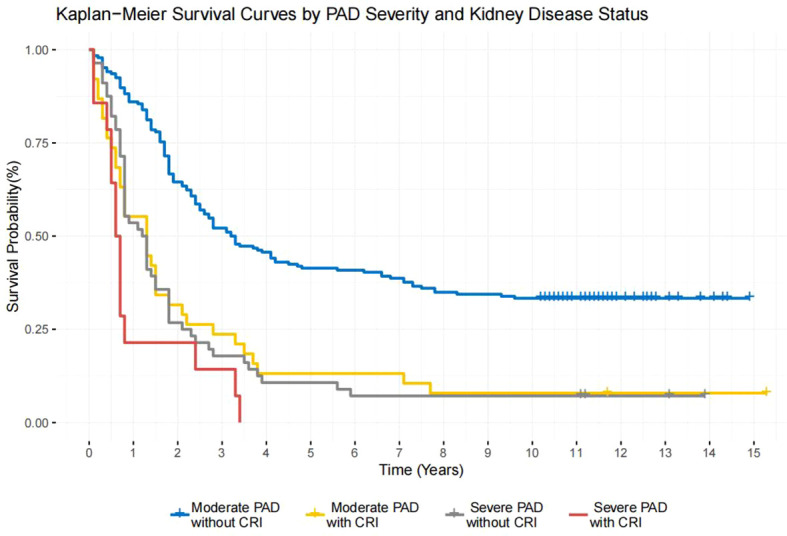
Kaplan−Meier survival curves by PAD severity and kidney disease status. Patients were stratified into: (1) Moderate PAD without CKD (reference group, ABI 0.41-0.69, n=186, blue), (2) Moderate PAD with CKD (ABI 0.41-0.69, n=38, yellow), (3) Severe PAD without CKD (ABI ≤0.4, n=56, gray), (4) Severe PAD with CKD (ABI ≤0.4, n=14, red). Significant survival differences were observed (Log-rank χ²=69.87, df=3, P<0.001). The severe PAD with CKD cohort showed the worst prognosis (3-year survival: 14.3% [95% CI 4.00-51.5] vs 52.2% [45.4-59.8] in moderate PAD without CKD; adjusted HR 5.52 [3.05-9.97], P<0.001). Censored data are marked with vertical ticks. Abbreviations: PAD, peripheral arterial disease; CKD, chronic kidney disease; ABI, ankle-brachial index.

To assess the combined impact of renal dysfunction and PAD on survival, we performed Kaplan-Meier (**Figure 1**) analysis stratified by CKD stage and severe PAD status (Log-rank P<0.001). The prognosis was profoundly poor for patients with both severe PAD and advanced CKD (G3-G5), exhibiting a median survival of only 0.65 years. In contrast, patients with neither condition had the most favorable outcome (median survival: 3.25 years). The observed synergistic mortality risk likely stems from the convergence of two distinct pathophysiological pathways: PAD exacerbates foot tissue ischemia and impairs wound healing, while CKD contributes to systemic uremia, accelerated cardiovascular disease, and immune dysfunction. This multiplicative effect highlights a critical high-risk phenotype in the DFU population.

### 3.5 Development of nomogram prediction model

**Figure 2:**
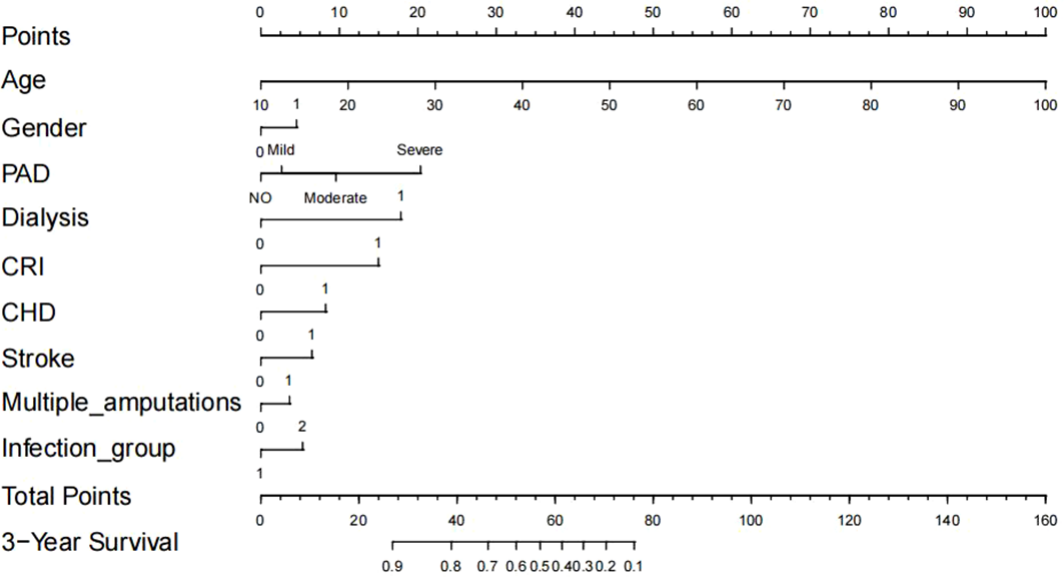
Development of the risk nomogram. Gender: 0 = Female, 1 = Male; PAD/Dialysis/CKD/CHD/Stroke/Multiple amputations: 0 = No, 1 = Yes; Infection group: 1 = Low, 2 = High.

Based on the nine significant risk factors identified in the model 1 of Cox multiple regression models, we developed a nomogram prediction model (**Figure 2**) to assess 3-year mortality risk in hospitalized diabetic foot patients. The model predictions demonstrated significant variation in the relative contributions of different risk factors to mortality risk. Notably, dialysis and CKD received high weighting scores, indicating their substantial impact on patient mortality. In contrast, factors such as gender showed lower weighting scores, reflecting their comparatively minor influence on mortality risk.

To use the nomogram: assign points for each variable, sum all points, and locate the total on the bottom scale to determine the 3-year survival probability. This estimate should complement, not replace, clinical judgment.

### 3.6 Validation of nomogram prediction model

**Figure 3:**
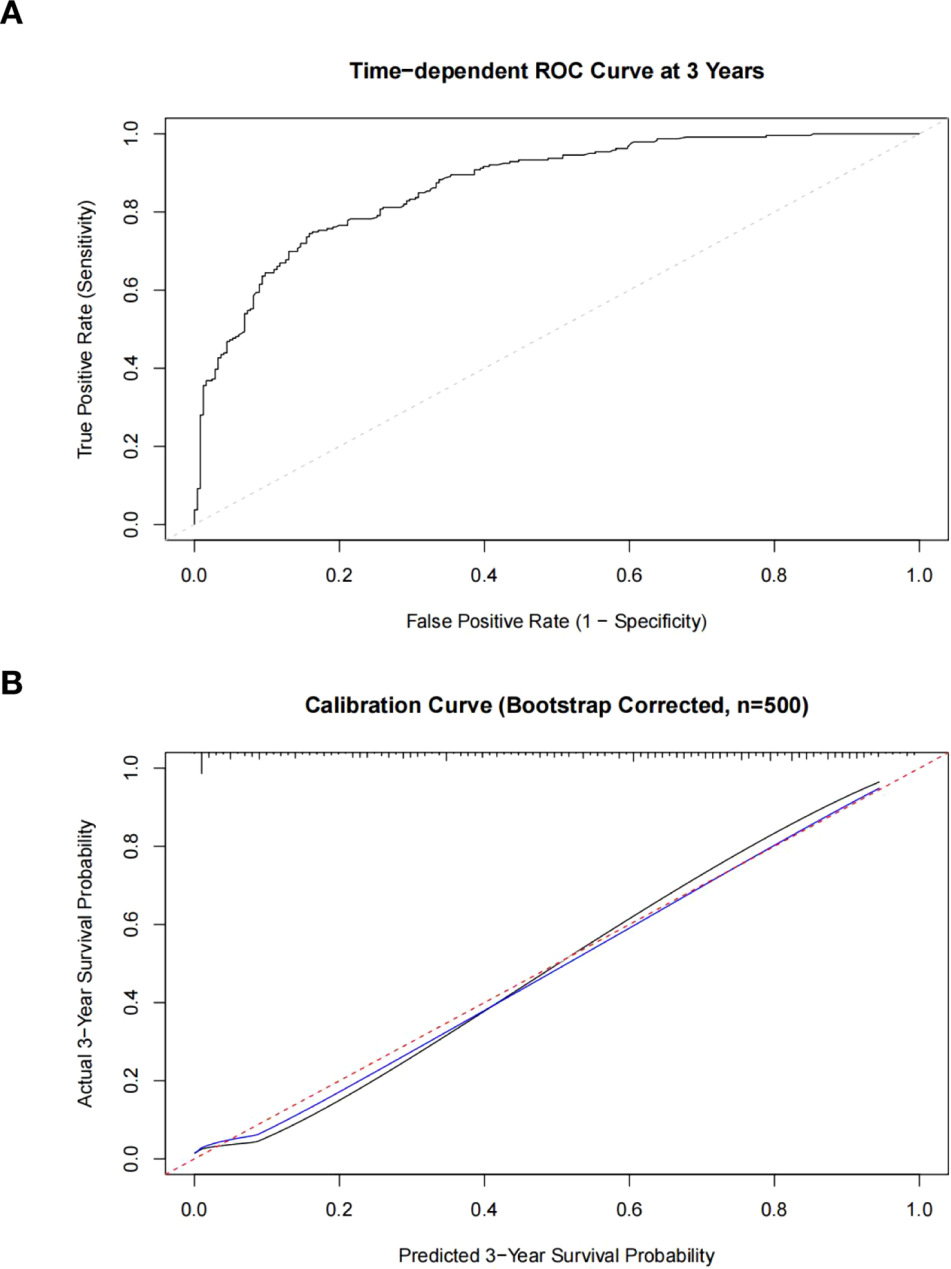
**(A)** The time-dependent ROC curve of the nomogram of the main mortality risk in patients with diabetic foot. Black solid line = Nomogram (3-Year AUC = 0.87, 95% CI 0.84–0.90); Gray dashed line = Random chance (AUC = 0.5). X-axis: 1 - Specificity; Y-axis: Sensitivity. Analysis by R package 'timeROC' using inverse probability of censoring weighting (IPCW). Abbreviations: AUC, area under the curve. **(B)** Calibration curve of the nomogram of the main mortality risk in patients with diabetic foot. Black solid line: Observed probabilities; Red dashed line: Ideal reference (45° line); Blue solid line: Optimism-corrected prediction (slope = 0.928). X-axis: Predicted 3−Year Survival Probability (%); Y-axis: Actual 3−Year Survival Probability (%). Bootstrap-corrected validation (500 repetitions) showed prediction error <5% in moderate-risk range (20-80%).

The nomogram prediction model developed in this study demonstrated excellent predictive performance through rigorous statistical validation. Model discrimination assessment revealed a Harrell’s C-index of 0.79 (95% CI: 0.77-0.81). Time-dependent ROC (**Figure 3A**) analysis at the 3-year endpoint yielded an AUC of 0.869 (95% CI: 0.84-0.90), confirming the model’s strong risk stratification capacity. Bootstrap internal validation (500 repetitions) resulted in an optimism-corrected calibration slope of 0.928, suggesting only minimal overfitting requiring adjustment with a shrinkage factor of 0.93. The calibration curve (**Figure 3B**) demonstrated excellent agreement between predicted and observed 3-year survival probabilities near the 45° reference line, with prediction error in the moderate-risk range (20-80%) maintained below 5%. Model performance analysis indicated a corrected R² of 0.474, explaining approximately 47.4% of outcome variation. Furthermore, the minimal difference in C-index between training and validation sets (Δ=0.014) confirmed the model’s robust generalizability and clinical utility.

## 4 Discussion

The study findings demonstrate that dialysis and CKD emerge as the most significant predictors of mortality risk in hospitalized diabetic foot patients. This aligns with previous studies, highlighting the critical role of renal dysfunction in determining patient prognosis (8, 10–12, 21, 22). Previous cohort studies have seldom incorporated the Wagner classification system directly into risk factor analyses. While infection-related markers such as WBC and CRP levels have been commonly employed as surrogate indicators, there has been a notable absence of systematic evaluations based on standardized diagnostic criteria, such as those outlined in the IDSA (Infectious Diseases Society of America classifications) guidelines. Furthermore, the severity of PAD significantly impacted patient survival, particularly among those with moderate to severe PAD, who exhibited a markedly increased mortality risk. This finding reinforces the importance of PAD as an independent predictor of cardiovascular events and mortality (23–25). Notably, in the second model, moderate and severe PAD remained significant (p < 0.05). These findings suggest a dose-response relationship between PAD severity and mortality risk, meaning that as PAD severity increases, patient mortality risk rises significantly.

Of note, several laboratory parameters reflective of glycemic control (HbA1c, FBG), anemia (Hb), dyslipidemia (TG, TC, HDL, LDL), systemic inflammation (CRP, WBC), and nutrition (ALB) were included in the initial univariate analysis due to their established pathophysiological roles in DFU progression and mortality. The markedly elevated median HbA1c level of 9.0% (IQR 7.6-10.5) observed in our cohort signifies poor long-term glycemic control, which is known to impair immune function, compromise wound healing, and promote microvascular complications (26). However, HbA1c did not retain independent significance in the final multivariable Cox model. This suggests that in this cohort with advanced disease, the prognostic influence of chronic hyperglycemia was likely superseded by the overwhelming impact of more advanced, life-threatening comorbidities such as end-stage renal disease requiring dialysis and severe peripheral arterial disease. Similarly, other laboratory markers, while potentially informative for specific pathophysiological processes (e.g., anemia reflecting compromised oxygen delivery, elevated CRP indicating systemic inflammation), were not selected into the final model. This does not negate their biological relevance but rather indicates that their predictive power for all-cause mortality was attenuated when adjusted for the dominant effects of irreversible end-organ damage in this high-risk population.

The Kaplan-Meier survival curve demonstrated that patients with both CKD and severe PAD had the lowest survival rate, approaching zero at 3-4 years, suggesting that the coexistence of these two risk factors may accelerate disease progression through multiple pathophysiological mechanisms. These results highlight the necessity of comprehensive management strategies for patients with comorbidities in clinical practice.

In this study, cardiovascular and cerebrovascular events accounted for more than half of the deaths, a finding that aligns entirely with data from other long-term follow-up studies conducted in Europe (7, 27, 28). In contrast, research from developing and newly industrialized countries indicates that a significantly higher proportion of deaths among patients with diabetic foot ulcers are attributed to septic diseases (2). A South Korean cohort study demonstrated a significant association between low socioeconomic status and increased mortality rates in diabetic foot patients (29). Existing clinical evidence supports that multidisciplinary team (MDT) approaches substantially improve clinical outcomes in this population (30). As China’s economic development has progressed, healthcare institutions have increasingly enhanced their capacity to provide comprehensive diabetic foot management. These factors may collectively contribute to the relatively lower proportion of infection-related mortality observed in our study compared to previous reports.

Infection group as an independent predictor of mortality underscores the significant impact of severe infection (Grade 3-4) on survival in patients with diabetic foot. Severe infection initiates a deleterious cycle at the wound site, impairing local tissue repair and promoting necrosis. More critically, it can precipitate a sustained systemic inflammatory response, characterized by the release of cytokines and acute-phase reactants, which may accelerate atherosclerosis, induce catabolism, and contribute to multi-organ dysfunction (16, 31). Unlike often irreversible conditions such as severe PAD and CKD, infection severity represents a potentially modifiable risk factor. This finding emphasizes the crucial importance of aggressive, multifaceted infection management—including timely debridement, targeted antimicrobial therapy, and metabolic control—as an essential strategy not only for limb salvage but also for improving overall survival.

Compared to previous studies, the 1-year, 3-year, and 5-year mortality rates of the target population in this study were significantly higher, the German cohort study reported mortality rates of 15.4% at 1-year follow-up, increasing to 33.1% at 3-years and 45.8% at 5-years (2, 32). This trend may be explained by the fact that this study was conducted at a single center, and all included cases were inpatients from tertiary grade A hospitals. Additionally, patients with milder cases of DFU were more likely to seek treatment at primary medical institutions.

Furthermore, multiple amputations have been identified as a significant risk factor influencing mortality in diabetic foot patients. Previous studies have primarily focused on the impact of major or minor amputations rather than multiple amputations (11, 33). Multiple amputations typically reflect the severity of the disease and the complexity of treatment, indicating that patients may have experienced recurrent infections, ischemic necrosis, or other complications. Our findings further demonstrate that patients with multiple amputations have a significantly higher mortality risk compared to those with a single amputation or no amputation (34). This suggests that the impact of amputation on mortality extends beyond the anatomical site of amputation itself. These findings emphasize the need to focus more closely on systemic, extra-foot conditions in patients with diabetic foot, as well as the cumulative burden of comorbidities associated with diabetes-related complications.

In the two Cox multiple regression models of this study, the reduction in the contribution of multiple amputations may reflect its multicollinearity or interaction with other risk factors. Multiple amputations are often closely associated with severe PAD and CKD, both of which have been established as independent predictors of mortality. Consequently, in the multivariate analysis, the impact of multiple amputations may have been overshadowed by the presence of PAD and CKD.

In recent years, the nomogram model has been widely utilized to predict clinical outcomes in patients with diabetic foot (13, 14). However, while existing studies have made significant strides in predicting risks such as amputation or ulcer healing, predictive models for mortality risk in diabetic foot patients remain relatively limited (35–37). This study developed a comprehensive nomogram incorporating multiple risk factors to assess mortality risk in hospitalized diabetic foot patients. Our analysis of **Figure 2** reveals that PAD exerts its most pronounced impact on mortality during intermediate-term follow-up (3-year horizon). The 3-year survival probability represents an optimal predictive window, effectively capturing peak risk while preventing model overfitting associated with long-term predictions (e.g., 5-10 years) due to data sparsity. Model performance was rigorously validated through time-dependent ROC analysis and calibration curve assessment (38, 39). The development and validation of our prognostic model were conducted in accordance with the Transparent Reporting of a multivariable prediction model for Individual Prognosis or Diagnosis (TRIPOD) statement (40). Notably, existing nomogram models for predicting amputation prognosis in diabetic foot patients share several risk factors with our model (e.g., PAD and CKD), while also incorporating additional potential predictors not included in our study (e.g., renal function-related indicators such as serum creatinine and blood urea nitrogen) (13, 35–37, 41, 42). Additionally, smoking history has been consistently identified as a significant risk factor in multiple diabetic foot prognostic models (35, 36, 41). While considered in our initial analysis, it was not retained in our final Cox multivariate regression and nomogram models. Future investigations should incorporate smoking history in prognostic analyses - even when not statistically significant in univariate testing - provided collinearity with other variables can be adequately controlled, given its established clinical relevance in diabetic complications (43, 44).

This study also has several limitations. First, the sample size is relatively limited, particularly the small number of dialysis patients, which may affect the stability of the results. Second, renal function-related indicators and family history, which are potential risk factors, were not considered (6, 10, 33). Additionally, while this study utilized single-center data, requiring multicenter validation to confirm generalizability, the tertiary care setting of our Grade A hospital in Eastern China enhances clinical relevance. Our patient cohort demonstrates representative regional and clinical characteristics, strengthening external validity. Future investigations should prioritize expanded sample sizes and multicenter collaboration to improve data reliability and translational significance. Furthermore, the current study did not comprehensively evaluate the etiological factors contributing to non-primary amputations in patients requiring multiple limb procedures, including potential associations with inadequate surgical radicality during initial amputations. This critical aspect warrants dedicated investigation in future research. Future research should focus on expanding the sample size and conducting multi-center studies to enhance reliability. Finally, the nomogram developed in this study underwent internal validation via Bootstrap resampling, which primarily assesses model stability within the original dataset but may not fully address overfitting risks. To ensure robust generalizability and clinical utility, future validation in independent multicenter cohorts is essential for confirming predictive performance across broader diabetic foot populations. The observed anemia in our cohort is likely multifactorial, but a significant contributor is presumably reduced erythropoietin synthesis secondary to the high prevalence of moderate-to-severe CKD, consistent with the known decline in production when eGFR falls below 45 mL/min/1.73 m². Unfortunately, red blood cell indices (e.g., MCV, MCH) were not available in our dataset to further characterize the anemia’s etiology. The inclusion of such parameters in future studies would be highly valuable to confirm this pathophysiological link and refine risk stratification.

In assessing the severity of diabetic foot, we employed the Wagner classification system. Although widely used in clinical practice, this system primarily focuses on ulcer depth and the degree of infection, with relatively limited evaluation of ischemic status. In contrast, the WIFi (Wound, Ischemia, and foot Infection) classification system integrates the three dimensions of wound, ischemia, and infection more comprehensively, offering greater advantages in classifying and predicting outcomes for diabetic foot patients (16, 45). Therefore, future research should consider adopting the WIFi classification system to provide a more comprehensive assessment of disease severity.

Second, when evaluating the severity of PAD, we primarily stratified patients based on the ABI. However, the accuracy of ABI can be compromised by arterial calcification, particularly in diabetic patients, where medial arterial calcification (MAC) is more prevalent. This can lead to artificially elevated ABI values, potentially masking the true severity of PAD and affecting the accuracy of stratification (46). Future studies should incorporate supplementary diagnostic methods, such as the toe-brachial index or imaging studies, to complement ABI assessments and mitigate the impact of arterial calcification on results (18).

## 5 Conclusion

In this study, dialysis and CKD emerged as the most significant predictors of mortality in patients with diabetic foot. When stratified by PAD, the impact of moderate and severe PAD on mortality was notably amplified. Additionally, we incorporated multiple amputations into the analysis and demonstrated their significant contribution to mortality risk. Combined analysis of causes of death and risk factors suggests that clinical management of diabetic foot should extend beyond local wound care to include comprehensive assessment and intervention for overall systemic health. Finally, we developed a nomogram model to evaluate 3-year mortality risks in patients with diabetic foot.

## Data Availability

The raw data supporting the conclusions of this article will be made available by the authors, without undue reservation.
